# Supplementary material for: Oleoylethanolamide Treatment Modulates Both Neuroinflammation and Microgliosis, and Prevents Massive Leukocyte Infiltration to the Cerebellum in a Mouse Model of Neuronal Degeneration
Source: Int J Mol Sci. 2023 Jun 2;24(11):9691. doi: 10.3390/ijms24119691 (PMC10253688; doi:10.3390/ijms24119691)

This document includes the following Supplementary Material:

- Figure S1: Graphs of the left hemi-cerebellum weight (a) and the proportion between the left hemi-cerebellum weight respect to the total body weight (b) in the short- (~P12), medium- (P20) and long-term (P30).
- Figure S2: Micrographs of detailed marker-based characterization of microglia in the cerebellum of untreated PCD and OEA-treated PCD mice at P30.
- Table S1: Comparisons and specific p-values from hemi-cerebellar weight and relation to the total body weight.
- Supplementary Information: Specificity and efficiency analyses of qPCR primers

Figure S1

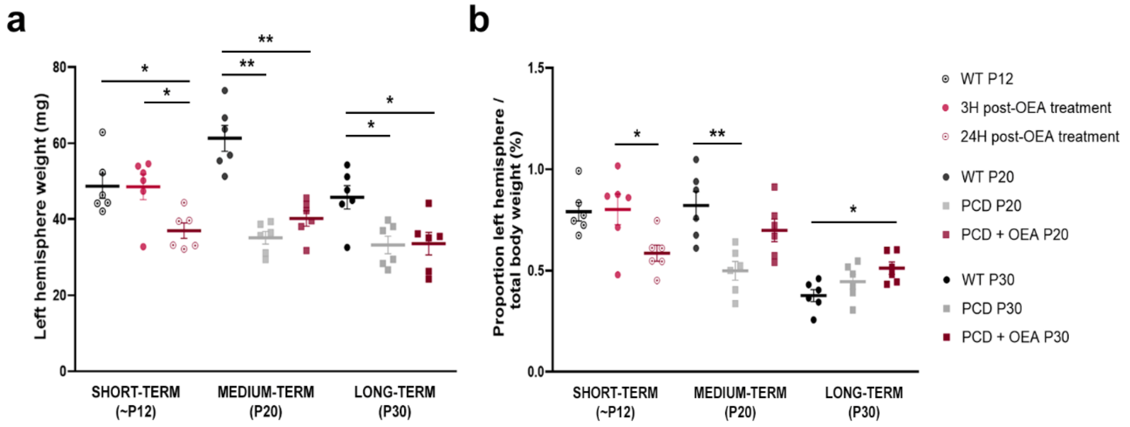

Figure S1. (a, b) Graphs of the left hemi-cerebellum weight (a) and the proportion between the left hemi-cerebellum weight respect to the total body weight (b) in the short- (~P12), medium- (P20) and long-term (P30). Analysis of left hemicerebellum weight revealed differences, in the short term, between WT untreated mice and WT mice 3 and 24 hours after OEA treatment, with the weight being lower in mice 24 hours after OEA treatment (WT: 49 ± 3 mg; 3 hours: 49 ± 3 mg; 24 hours: 37 ± 2 mg). At P20 and P30 (i.e., medium and long term) differences were found between WT animals and the two experimental groups of PCD mice, with the weight of the mutants being less (P20, WT: 61 ± 3 mg, PCD: 35 ± 1 mg, PCD+OEA: 40 ± 2 mg; P30, WT: 45 ± 3 mg, PCD: 33 ± 2 mg, PCD+OEA: 33 ± 3 mg). However, when the ratio of hemicerebellum weight to total body weight was analyzed, differences were only detected between the groups 3 and 24 hours after treatment in the short term, with the percentage being lower after 24 hours (WT: 0.79 ± 0.04%, 3 hours: 0.80 ± 0.07%, 24 hours: 0.58 ± 0.04%). Regarding the medium term, differences were found between WT and untreated PCD mice, the percentage being lower in mutants compared to the wild type (WT: 0.82 ± 0.06%, PCD: 0.49 ± 0.04%, PCD+OEA: 0.69 ± 0.05%). Concerning long-term analysis, differences were limited to WT *vs.* OEA-treated PCD mice at P30, with the percentage being higher in those PCD mice treated with OEA (WT: 0.37 ± 0.02%, PCD: 0.44 ± 0.03%, PCD+OEA: 0.51 ± 0.03%). Due to the non-anorexigenic effect on the total body weight reported for the dose of OEA used in our study (Pérez-Martín, *et al.*, 2021), these results suggest that the weight of the hemicerebellum of OEA-treated PCD mice was greater than their total body weight; that is, cerebellar atrophy in these treated animals was lower than in the untreated mice. n=6 animals per experimental group. One-way ANOVA followed by Bonferroni's post hoc test. * $p < 0.05$; ** $p < 0.01$

Figure S2

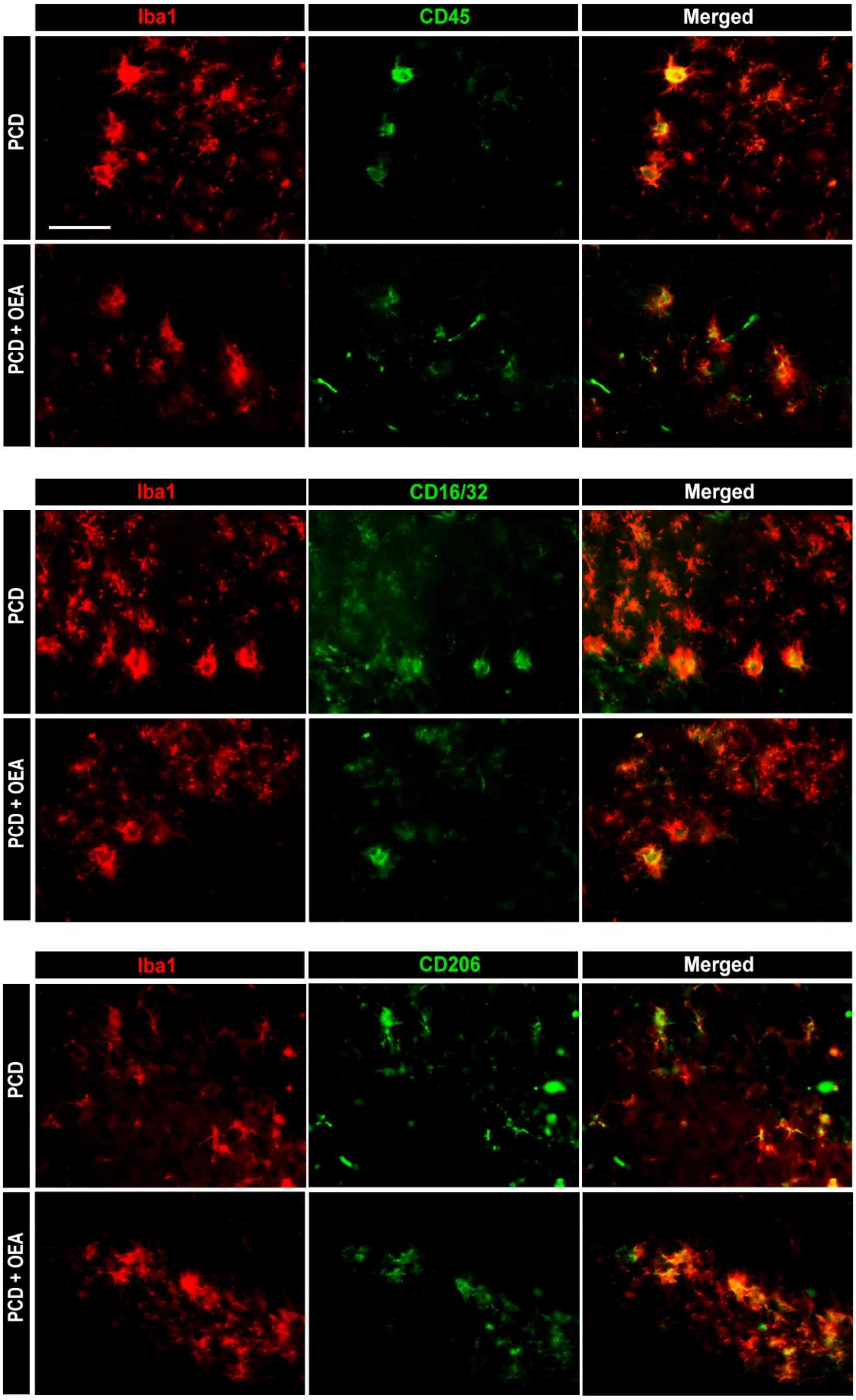

Figure S2. Micrograph of detailed marker-based characterization of microglia in the cerebellum of untreated PCD and OEA-treated PCD mice at P30. At the top, immunolabeling for Iba1 (red) and CD45 (green), i.e., reactive microglial cells. In the center, immunolabeling for Iba1 (red) and CD16/32

(green), i.e., proinflammatory microglial cells. At the bottom, immunolabeling for Iba1 (red) and CD206 (green), i.e., neuroprotective or tissue reparative microglial cells. Scale bar: 20 μm.

Table S1. Comparisons and specific *p*-values from hemi-cerebellar weight and relation to the total body weight.

Variables	Comparisons	*p*-values
Hemi-cerebellar weight ~P12-P13	WT *vs.* WT 3H Post-OEA	1.000
	WT *vs.* WT 24H Post-OEA	**0.036**
	WT 3H Post-OEA *vs.* WT 24H Post-OEA	**0.039**
Hemi-cerebellar weight P20	WT *vs.* PCD	**< 0.001**
	WT *vs.* PCD+OEA	**< 0.001**
	PCD *vs.* PCD+OEA	0.521
Hemi-cerebellar weight P30	WT *vs.* PCD	**0.019**
	WT *vs.* PCD+OEA	**0.023**
	PCD *vs.* PCD+OEA	1.000
Hemi-cerebellum/ Total Body weight ~P12-P13	WT *vs.* 3H Post-OEA	1.000
	WT *vs.* WT 24H Post-OEA	0.061
	WT 3H Post-OEA vs. WT 24H Post-OEA	**0.046**
Hemi-cerebellum/ Total Body weight P20	WT *vs.* PCD	**0.004**
	WT *vs.* PCD+OEA	0.451
	PCD vs. PCD+OEA	0.081
Hemi-cerebellum/Total Body weight P30	WT *vs.* PCD	0.472
	WT *vs.* PCD+OEA	**0.029**
	PCD *vs.* PCD+OEA	0.477

Bold values indicate statistical significance at the $p < 0.05$.

Supplementary Information. Specificity and efficiency analyses of qPCR primers

**Gene**	***Bdnf***
MGI/NCBI ID	88145/12064
Forward primer	GTGGTGTAAGCCGCAAAGA
Reverse primer	AACCATAGTAAGGAAAAGGATGGTC

Job name: Job 2: RBDNF_1L
Species: Mouse (Mus musculus)
Assembly: GRCm38
Search type: BLASTN (NCBI Blast)

Download results file | New job

Results table

Show All entries | Show/hide columns (2 hidden) | Filter

Genomic Location	Overlapping Gene(s)	Orientation	Query start	Query end	Length	Score	E-val	%ID
2:109723405-109723429 [Sequence]	Bdnf	Reverse	1	25	25 [Sequence]	49.9	7e-05	100.00 [Alignment]
X:115466059-115466076 [Sequence]		Reverse	4	21	18 [Sequence]	36.1	1.0	100.00 [Alignment]
16:59726024-59726041 [Sequence]	Epha6	Reverse	4	21	18 [Sequence]	36.1	1.0	100.00 [Alignment]

Job name: Job 1: FBDNF_1L
Species: Mouse (Mus musculus)
Assembly: GRCm38
Search type: BLASTN (NCBI Blast)

Download results file | New job

Results table

Show All entries | Show/hide columns (2 hidden) | Filter

Genomic Location	Overlapping Gene(s)	Orientation	Query start	Query end	Length	Score	E-val	%ID
2:109676375-109676393 [Sequence]	Gm45346, Bdnf	Forward	1	19	19 [Sequence]	38.1	0.087	100.00 [Alignment]
X:145325531-145325549 [Sequence]	Lhfpl1	Forward	1	19	19 [Sequence]	30.2	21	94.74 [Alignment]
17:44550474-44550488 [Sequence]	Runx2	Reverse	5	19	15 [Sequence]	30.2	21	100.00 [Alignment]

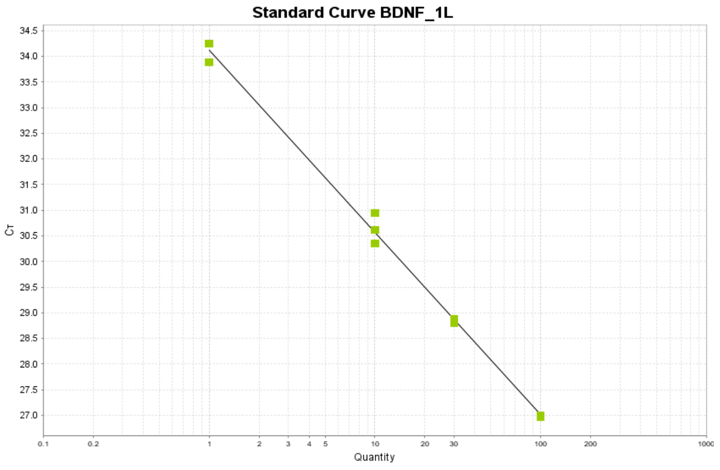

Target: BDNF_L Slope: -3.553 Y-Inter: 34.115 R^2: 0.996 Eff%: 91.181 Error: 0.076

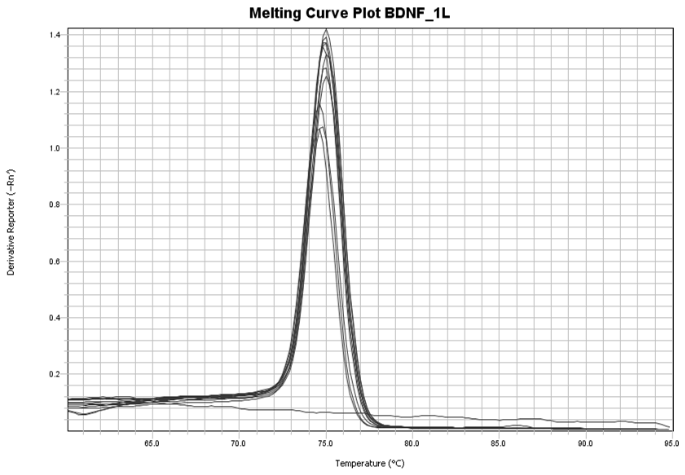

Gene	*Cox2 (Ptgs2)*
MGI/NCBI ID	977798/19225
Forward primer	GGTCATTGGTGGAGAGGTGTA
Reverse primer	TGAGTCTGCTGGTTTGGAATAG

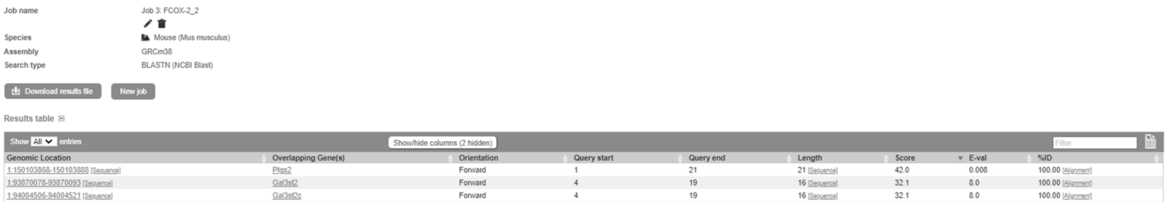

Job name: Job 3: FCOX-2_2
Species: Mouse (Mus musculus)
Assembly: GRCm38
Search type: BLASTN (NCBI Blast)

Download results file | New job

Results table

Show All entries | Show/hide columns (2 hidden) | Filter

Genomic Location	Overlapping Gene(s)	Orientation	Query start	Query end	Length	Score	E-val	%ID
1:150103868-150103888 [Sequence]	Ptgs2	Forward	1	21	21 [Sequence]	42.0	0.008	100.00 [Alignment]
1:93870078-93870093 [Sequence]	Gal3st2	Forward	4	19	16 [Sequence]	32.1	8.0	100.00 [Alignment]
1:94004506-94004521 [Sequence]	Gal3st2c	Forward	4	19	16 [Sequence]	32.1	8.0	100.00 [Alignment]

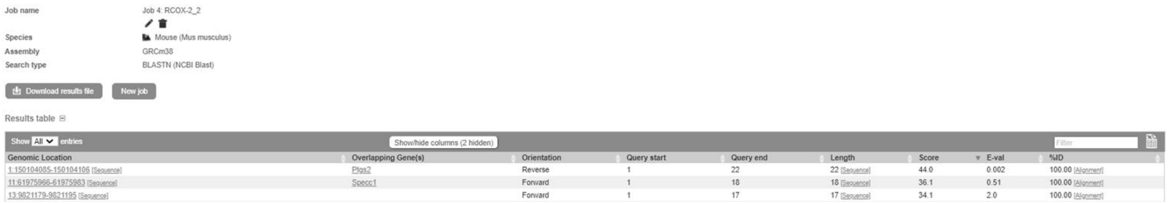

Job name: Job 4: RCOX-2_2
Species: Mouse (Mus musculus)
Assembly: GRCm38
Search type: BLASTN (NCBI Blast)

Download results file | New job

Results table

Show All entries | Show/hide columns (2 hidden) | Filter

Genomic Location	Overlapping Gene(s)	Orientation	Query start	Query end	Length	Score	E-val	%ID
1:150104085-150104106 [Sequence]	Ptgs2	Reverse	1	22	22 [Sequence]	44.0	0.002	100.00 [Alignment]
11:61975966-61975983 [Sequence]	Specc1	Forward	1	18	18 [Sequence]	36.1	0.51	100.00 [Alignment]
13:9821179-9821195 [Sequence]		Forward	1	17	17 [Sequence]	34.1	2.0	100.00 [Alignment]

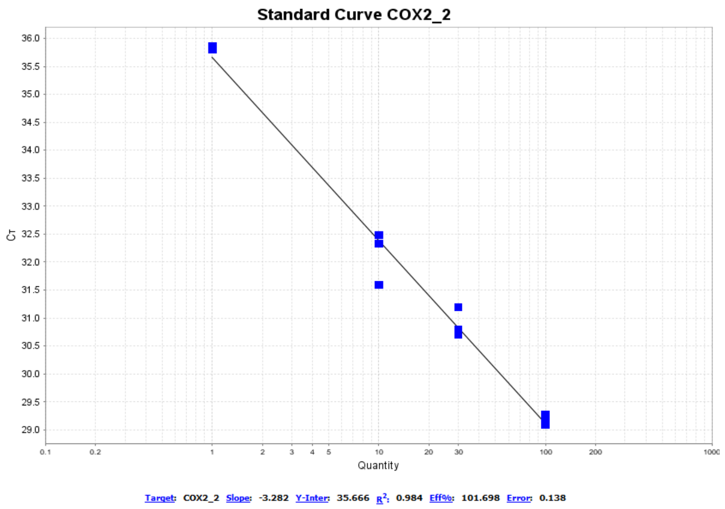

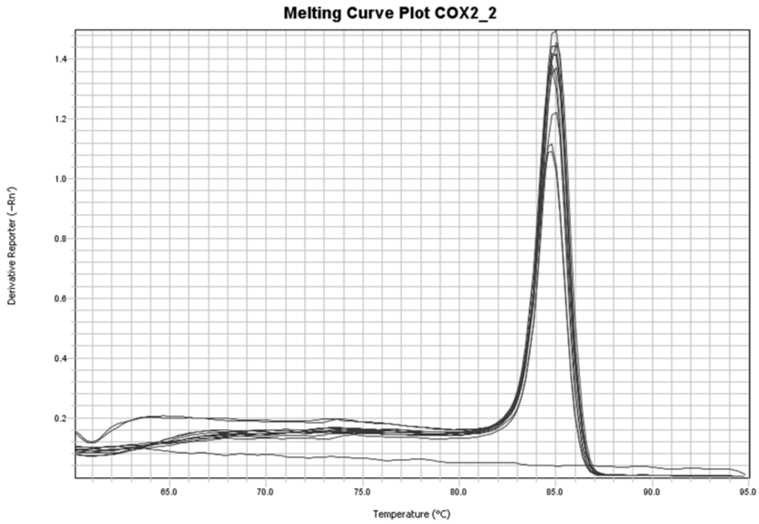

Gene	*Gap43*
MGI/NCBI ID	95639/14432
Forward primer	GCTGGTGCATCACCCTTCT
Reverse primer	TGGTGTCAAGCCGGAAGATAA

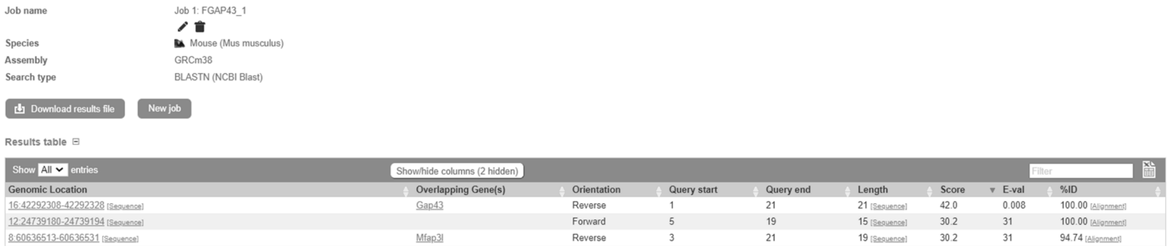

Job name Job 1: FGAP43_1

Species Mouse (Mus musculus)

Assembly GRCm38

Search type BLASTN (NCBI Blast)

Download results file New job

Results table

Show All entries Show/hide columns (2 hidden) Filter

Genomic Location	Overlapping Gene(s)	Orientation	Query start	Query end	Length	Score	E-val	%ID
16:42292308-42292328 [Sequence]	Gap43	Reverse	1	21	21 [Sequence]	42.0	0.008	100.00 [Alignment]
12:24739180-24739194 [Sequence]		Forward	5	19	15 [Sequence]	30.2	31	100.00 [Alignment]
8:60636513-60636531 [Sequence]	Mfap3l	Reverse	3	21	19 [Sequence]	30.2	31	94.74 [Alignment]

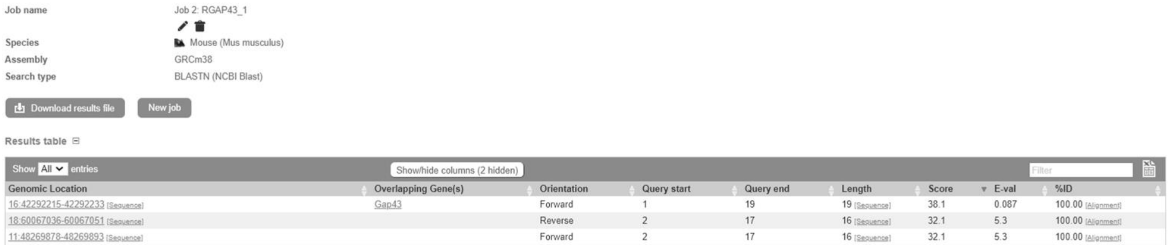

Job name Job 2: RGAP43_1

Species Mouse (Mus musculus)

Assembly GRCm38

Search type BLASTN (NCBI Blast)

Download results file New job

Results table

Show All entries Show/hide columns (2 hidden) Filter

Genomic Location	Overlapping Gene(s)	Orientation	Query start	Query end	Length	Score	E-val	%ID
16:42292215-42292233 [Sequence]	Gap43	Forward	1	19	19 [Sequence]	38.1	0.087	100.00 [Alignment]
18:60067036-60067051 [Sequence]		Reverse	2	17	16 [Sequence]	32.1	5.3	100.00 [Alignment]
11:48269878-48269893 [Sequence]		Forward	2	17	16 [Sequence]	32.1	5.3	100.00 [Alignment]

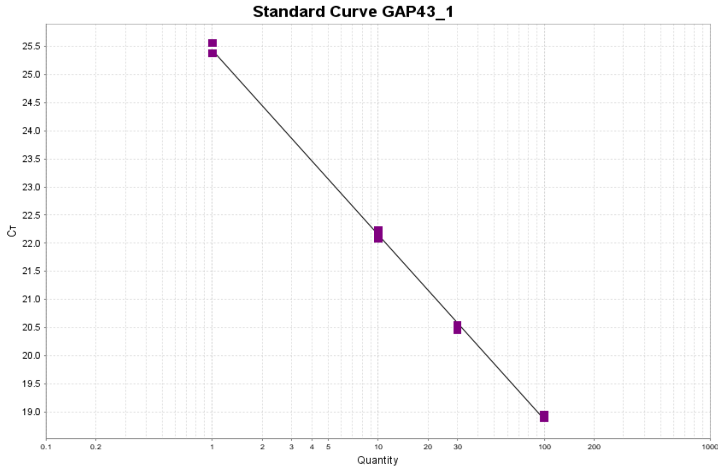

Target: GAP43_1 Slope: -3.293 Y-Inter: 25.44 R^2: 0.999 Eff%: 101.234 Error: 0.038

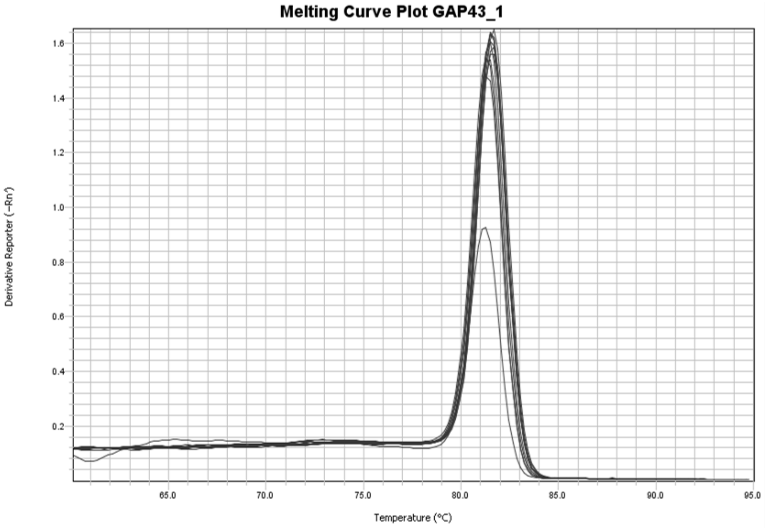

Gene	*Ifnγ*
MGI/NCBI ID	107656/15978
Forward primer	CAGCAACAGCAAGGCGAAAAAGG
Reverse primer	TTTCCGCTTCCTGAGGCTGGAT

Job name: Job 3: F IFNg_5
Species: Mouse (Mus musculus)
Assembly: GRCm38
Search type: BLASTN (NCBI Blast)

Download results file | New job

Results table

Show All entries | Show/hide columns (2 hidden) | Filter

Genomic Location	Overlapping Gene(s)	Orientation	Query start	Query end	Length	Score	E-val	%ID
10:118442744-118442766 [Sequence]	Ifng, Gm49751	Forward	1	23	23 [Sequence]	46.0	7e-04	100.00 [Alignment]
6:88084544-88084560 [Sequence]	Rpn1	Reverse	1	17	17 [Sequence]	34.1	2.7	100.00 [Alignment]
6:122602286-122602301 [Sequence]	Apobec1	Reverse	1	16	16 [Sequence]	32.1	11	100.00 [Alignment]

Job name: Job 4: R IFNg_5
Species: Mouse (Mus musculus)
Assembly: GRCm38
Search type: BLASTN (NCBI Blast)

Download results file | New job

Results table

Show All entries | Show/hide columns (2 hidden) | Filter

Genomic Location	Overlapping Gene(s)	Orientation	Query start	Query end	Length	Score	E-val	%ID
10:118445225-118445246 [Sequence]	Ifng	Reverse	1	22	22 [Sequence]	44.0	0.002	100.00 [Alignment]
15:97777534-97777550 [Sequence]		Forward	6	22	17 [Sequence]	34.1	2.0	100.00 [Alignment]
7:127866783-127866799 [Sequence]	Zfp668	Forward	6	22	17 [Sequence]	34.1	2.0	100.00 [Alignment]

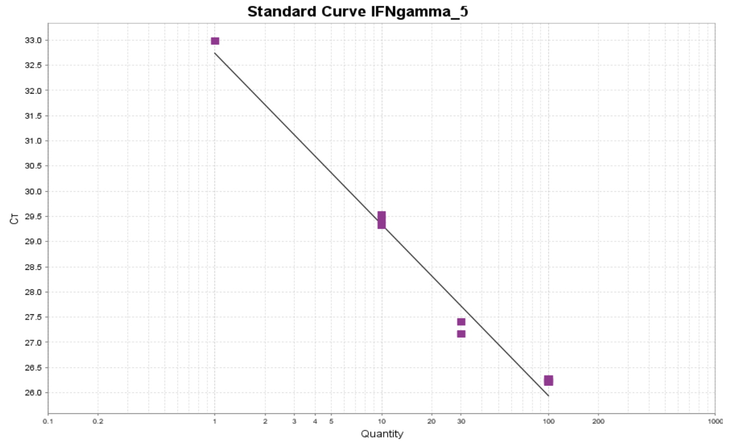

Target: PPARa_6 Slope: -3.403 Y-Inter: 32.74 R^2: 0.974 Eff%: 96.709 Error: 0.196

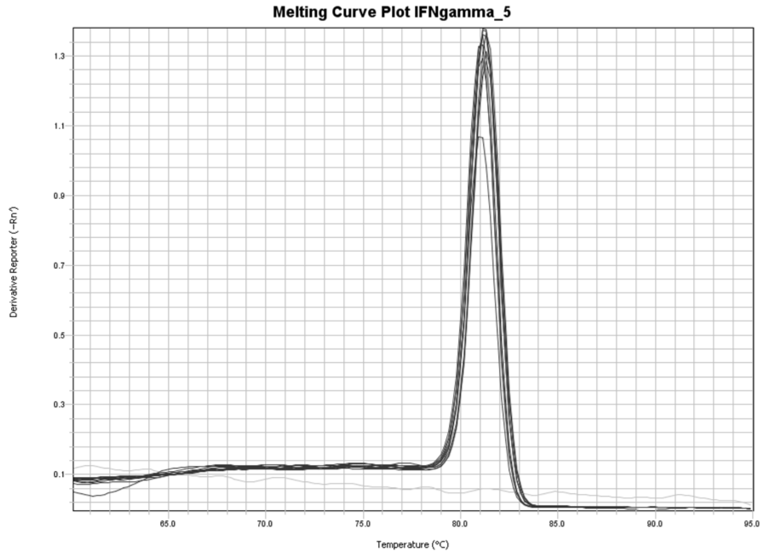

Gene	*Il1β*
MGI/NCBI ID	96543/16176
Forward primer	TGCTCATGTCCTCATCCTGGAAGG
Reverse primer	TCGCAGCAGCACATCAACAAGAG

Job name Job 3: FIL-1B_2
Species Mouse (Mus musculus)
Assembly GRCm38
Search type BLASTN (NCBI Blast)

Download results file | New job

Results table

Show All entries | Show/hide columns (2 hidden) | Filter

Genomic Location	Overlapping Gene(s)	Orientation	Query start	Query end	Length	Score	E-val	%ID
2:129368079-129368101 [Sequence]	Il1b	Reverse	1	23	23 [Sequence]	46.0	7e-04	100.00 [Alignment]
CHR_MG4198_PATCH:7486517-7486532 [Sequence]		Reverse	8	23	16 [Sequence]	32.1	11	100.00 [Alignment]
17:7535084-7535099 [Sequence]		Reverse	8	23	16 [Sequence]	32.1	11	100.00 [Alignment]

Job name Job 4: RIL-1B_2
Species Mouse (Mus musculus)
Assembly GRCm38
Search type BLASTN (NCBI Blast)

Download results file | New job

Results table

Show All entries | Show/hide columns (2 hidden) | Filter

Genomic Location	Overlapping Gene(s)	Orientation	Query start	Query end	Length	Score	E-val	%ID
2:129367984-129368007 [Sequence]	Il1b	Forward	1	24	24 [Sequence]	48.0	2e-04	100.00 [Alignment]
2:24873612-24873628 [Sequence]	Ehmt1	Forward	6	22	17 [Sequence]	34.1	3.4	100.00 [Alignment]
2:50624565-50624581 [Sequence]		Forward	3	19	17 [Sequence]	34.1	3.4	100.00 [Alignment]

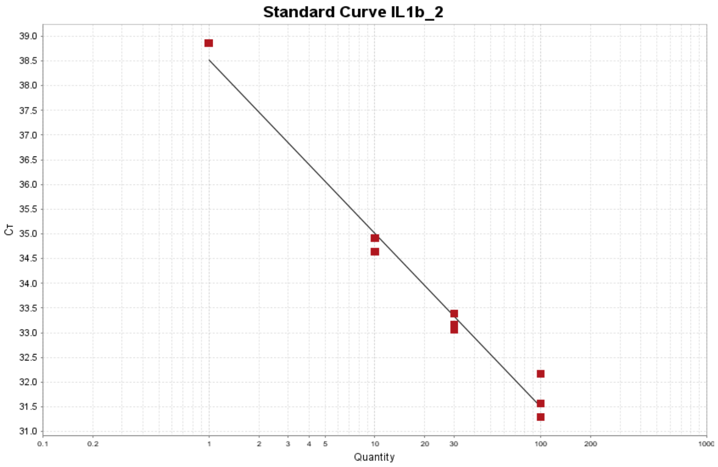

Target: IL1B_2 Slope: -3.51 Y-Inter: 38.52 R^2: 0.979 Eff%: 92.708 Error: 0.192

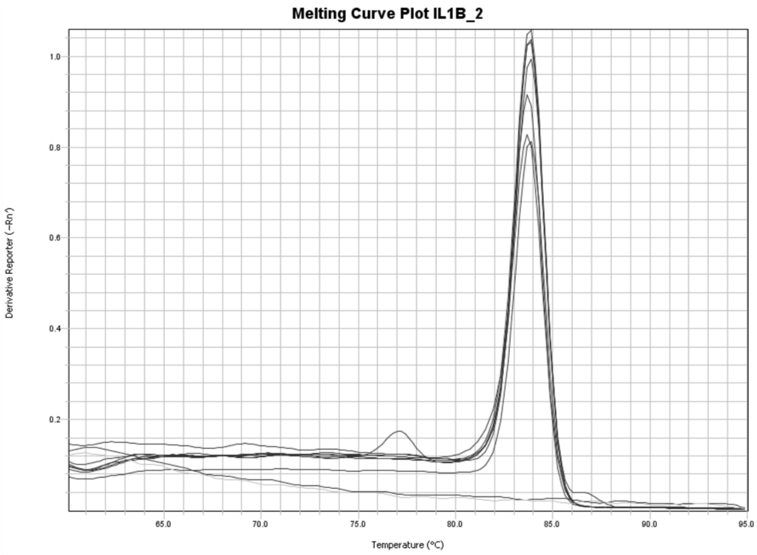

Gene	*Il6*
MGI/NCBI ID	96559/16193
Forward primer	GAGGATACCACTCCCAACAGACC
Reverse primer	AAGTGCATCATCGTTGTTCATACA

Job name: Job 5: FIL-6_3
Species: Mouse (Mus musculus)
Assembly: GRCm38
Search type: BLASTN (NCBI Blast)

Download results file | New job

Results table

Show All entries | Show/hide columns (2 hidden) | Filter

Genomic Location	Overlapping Gene(s)	Orientation	Query start	Query end	Length	Score	E-val	%ID
5:30013465-30013487 [Sequence]	Il6	Forward	1	23	23 [Sequence]	46.0	7e-04	100.00 [Alignment]
7:133653834-133653851 [Sequence]	Edrf1	Reverse	6	23	18 [Sequence]	36.1	0.69	100.00 [Alignment]
CHR_MG3530_PATCH:67271492-67271508 [Sequence]	Nrbf2	Forward	3	19	17 [Sequence]	34.1	2.7	100.00 [Alignment]

Job name: Job 6: RIL-6_3
Species: Mouse (Mus musculus)
Assembly: GRCm38
Search type: BLASTN (NCBI Blast)

Download results file | New job

Results table

Show All entries | Show/hide columns (2 hidden) | Filter

Genomic Location	Overlapping Gene(s)	Orientation	Query start	Query end	Length	Score	E-val	%ID
5:30014853-30014876 [Sequence]	Il6	Reverse	1	24	24 [Sequence]	48.0	2e-04	100.00 [Alignment]
16:16197868-16197883 [Sequence]		Forward	6	21	16 [Sequence]	32.1	13	100.00 [Alignment]
3:71623326-71623341 [Sequence]		Reverse	9	24	16 [Sequence]	32.1	13	100.00 [Alignment]

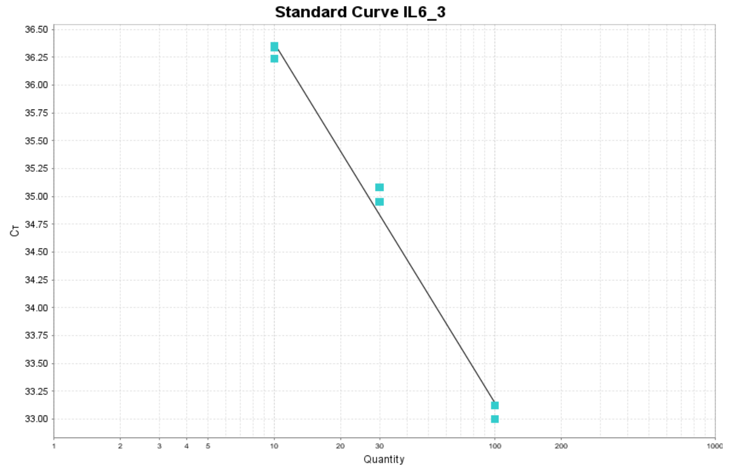

Target: IL6_3 Slope: -3.229 Y-Inter: 39.603 R^2: 0.991 Eff%: 104.043 Error: 0.14

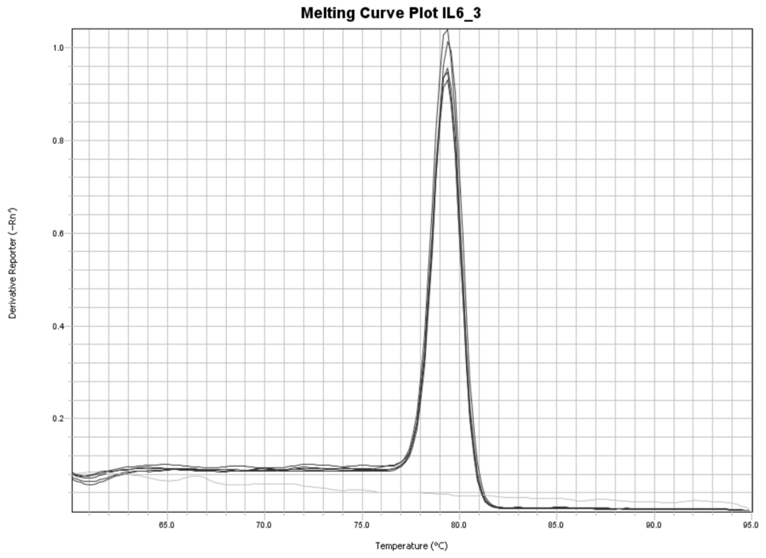

Gene	*iNos (Nos2)*
MGI/NCBI ID	97361/18126
Forward primer	CTTTGCCACGGACGAGAC
Reverse primer	AACTTCCAGTCATTGTACTCTGAGG

Job name Job 1: FiNOS_D

Species Mouse (Mus musculus)
Assembly GRCm38
Search type BLASTN (NCBI Blast)

Download results file New job

Results table

Show All entries Show/hide columns (2 hidden) Filter

Genomic Location	Overlapping Gene(s)	Orientation	Query start	Query end	Length	Score	E-val	%ID
11:78951210-78951227 [Sequence]	Nos2	Forward	1	18	18 [Sequence]	36.1	0.34	100.00 [Alignment]
5:37554037-37554051 [Sequence]	Stk32b	Forward	4	18	15 [Sequence]	30.2	21	100.00 [Alignment]
3:17677861-17677874 [Sequence]		Reverse	5	18	14 [Sequence]	28.2	82	100.00 [Alignment]

Job name Job 2: RiNOS_D

Species Mouse (Mus musculus)
Assembly GRCm38
Search type BLASTN (NCBI Blast)

Download results file New job

Results table

Show All entries Show/hide columns (2 hidden) Filter

Genomic Location	Overlapping Gene(s)	Orientation	Query start	Query end	Length	Score	E-val	%ID
11:78952804-78952828 [Sequence]	Nos2	Reverse	1	25	25 [Sequence]	49.9	7e-05	100.00 [Alignment]
9:25533893-25533910 [Sequence]	Eepd1	Forward	8	25	18 [Sequence]	36.1	1.0	100.00 [Alignment]
X:99111652-99111673 [Sequence]		Reverse	2	22	22 [Sequence]	34.1	4.0	95.45 [Alignment]

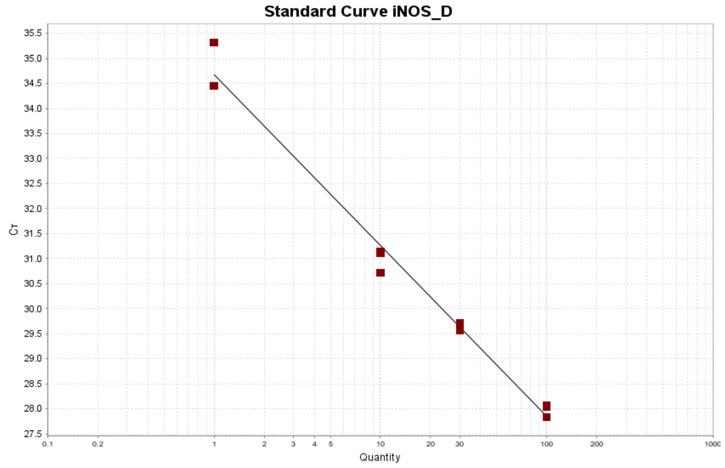

Target: INOS_D Slope: -3.417 Y-Inter: 34.677 R^2: 0.985 Eff%: 96.168 Error: 0.138

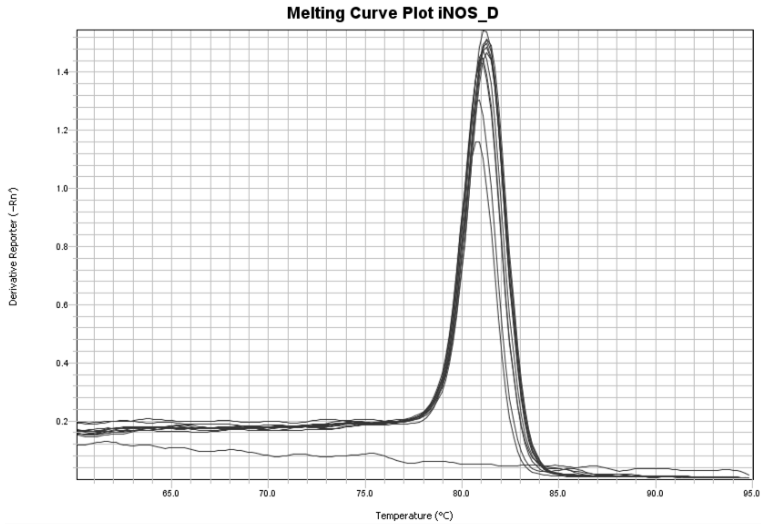

Gene	*Map2*
MGI/NCBI ID	97175/17756
Forward primer	GCTGTAGCAGTCCTGAAAGGTG
Reverse primer	CTTCCTCCACTGTGGCTGTTTG

Job name Job 1: F MAP2_8

Species Mouse (Mus musculus)

Assembly GRCm38

Search type BLASTN (NCBI Blast)

Download results file | New job

Results table

Show All entries | Show/hide columns (2 hidden) | Filter

Genomic Location	Overlapping Gene(s)	Orientation	Query start	Query end	Length	Score	E-val	%ID
1:66399430-66399451 [Sequence]	Map2	Forward	1	22	22 [Sequence]	44.0	0.002	100.00 [Alignment]
13:109058951-109058967 [Sequence]	Pde4d	Forward	4	20	17 [Sequence]	34.1	2.0	100.00 [Alignment]
5:51897597-51897613 [Sequence]		Forward	4	20	17 [Sequence]	34.1	2.0	100.00 [Alignment]

Job name Job 2: R MAP2_8

Species Mouse (Mus musculus)

Assembly GRCm38

Search type BLASTN (NCBI Blast)

Download results file | New job

Results table

Show All entries | Show/hide columns (2 hidden) | Filter

Genomic Location	Overlapping Gene(s)	Orientation	Query start	Query end	Length	Score	E-val	%ID
1:66401500-66401521 [Sequence]	Map2	Reverse	1	22	22 [Sequence]	44.0	0.002	100.00 [Alignment]
7:110892973-110892990 [Sequence]	Mrvi1	Reverse	1	18	18 [Sequence]	36.1	0.51	100.00 [Alignment]
5:127433533-127433550 [Sequence]	Tmem132c	Forward	1	18	18 [Sequence]	36.1	0.51	100.00 [Alignment]

Standard Curve MAP2_8

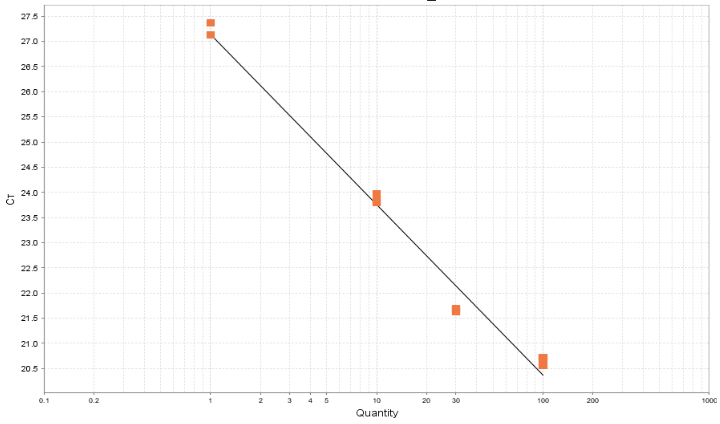

Target: MAP2_8 Slope: -3.39 Y-Inter: 27.143 R^2: 0.982 Eff%: 97.247 Error: 0.151

Melting Curve Plot MAP2_8

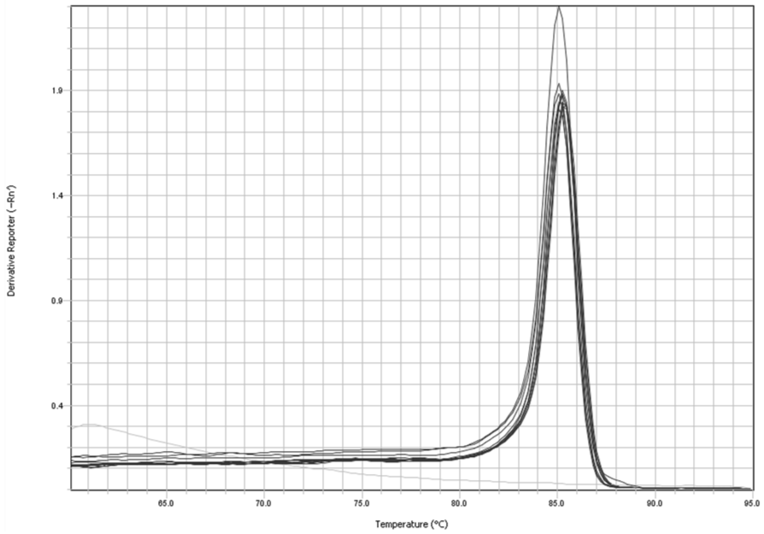

Gene	*Nfκb*
MGI/NCBI ID	1914300/67050
Forward primer	GCTGCCAAAGAAGGACACGACA
Reverse primer	GGCAGGCTATTGCTCATCACAG

Job name: Job 5: F NFKB_3
Species: Mouse (Mus musculus)
Assembly: GRCm38
Search type: BLASTN (NCBI Blast)

Download results file | New job

Results table

Show All entries | Show/hide columns (2 hidden)

Genomic Location	Overlapping Gene(s)	Orientation	Query start	Query end	Length	Score	E-val	%ID
3:135594931-135594952 [Sequence]	Nfkb1	Reverse	1	22	22 [Sequence]	44.0	0.002	100.00 [Alignment]
12:84380205-84380221 [Sequence]	Entpd5	Reverse	1	17	17 [Sequence]	34.1	2.0	100.00 [Alignment]
11:82735770-82735786 [Sequence]	Cct6b	Forward	2	18	17 [Sequence]	34.1	2.0	100.00 [Alignment]

Job name: Job 6: R NFKB_3
Species: Mouse (Mus musculus)
Assembly: GRCm38
Search type: BLASTN (NCBI Blast)

Download results file | New job

Results table

Show All entries | Show/hide columns (2 hidden)

Genomic Location	Overlapping Gene(s)	Orientation	Query start	Query end	Length	Score	E-val	%ID
3:135594336-135594357 [Sequence]	Nfkb1	Forward	1	22	22 [Sequence]	44.0	0.002	100.00 [Alignment]
9:112092346-112092362 [Sequence]	Arpp21	Forward	6	22	17 [Sequence]	34.1	2.0	100.00 [Alignment]
3:7679587-7679602 [Sequence]	Gm16685	Reverse	5	20	16 [Sequence]	32.1	8.0	100.00 [Alignment]

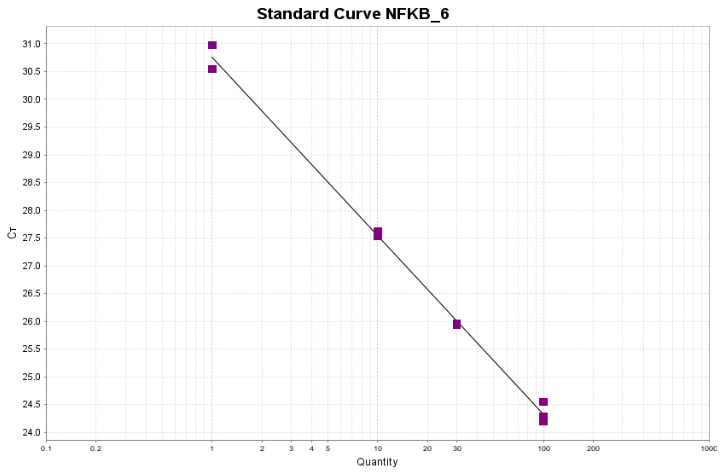

Target: NFKB_3 Slope: -3.223 Y-Inter: 30.764 R^2: 0.997 Eff%: 104.31 Error: 0.062

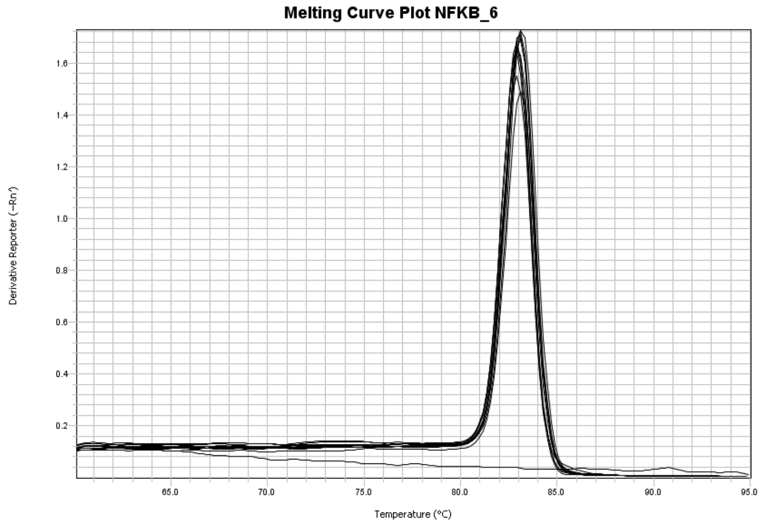

Gene	*Ppara*
MGI/NCBI ID	104740/19013
Forward primer	ATGCCAGTACTGCCGTTTTC
Reverse primer	TTGCCCAGAGATTTGAGGTC

Job name: Job 9: FPPAR-alpha_6
Species: Mouse (Mus musculus)
Assembly: GRCm38
Search type: BLASTN (NCBI Blast)

Download results file | New job

Results table

Show All entries — Show/hide columns (2 hidden) — Filter

Genomic Location	Overlapping Gene(s)	Orientation	Query start	Query end	Length	Score	E-val	%ID
15:85789065-85789084 [Sequence]	Ppara	Forward	1	20	20 [Sequence]	40.0	0.022	100.00 [Alignment]
1:37498865-37498880 [Sequence]	Mgat4a	Forward	5	20	16 [Sequence]	32.1	5.3	100.00 [Alignment]
1:182904643-182904657 [Sequence]		Forward	1	15	15 [Sequence]	30.2	21	100.00 [Alignment]

Job name: Job 10: RPPAR-alpha_6
Species: Mouse (Mus musculus)
Assembly: GRCm38
Search type: BLASTN (NCBI Blast)

Download results file | New job

Results table

Show All entries — Show/hide columns (2 hidden) — Filter

Genomic Location	Overlapping Gene(s)	Orientation	Query start	Query end	Length	Score	E-val	%ID
15:85790935-85790954 [Sequence]	Ppara	Reverse	1	20	20 [Sequence]	40.0	0.022	100.00 [Alignment]
16:9154006-9154022 [Sequence]		Reverse	3	19	17 [Sequence]	34.1	1.3	100.00 [Alignment]
12:28453633-28453649 [Sequence]	Dcdc2c	Reverse	1	17	17 [Sequence]	34.1	1.3	100.00 [Alignment]

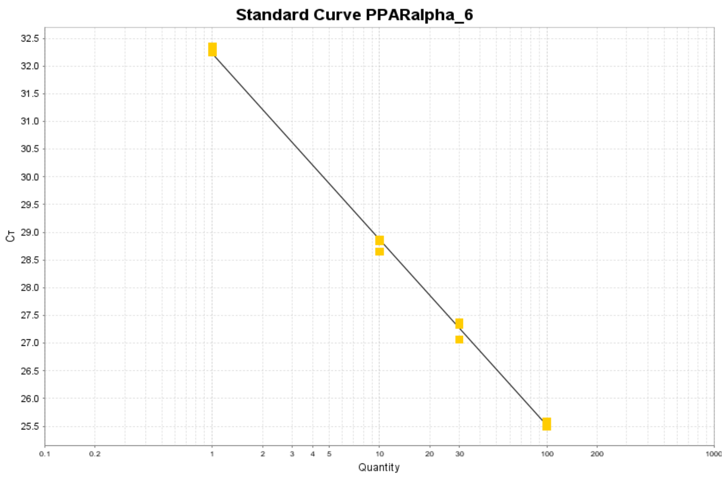

Target: PPARa_6 Slope: -3.362 Y-Inter: 32.23 R^2: 0.998 Eff%: 98.346 Error: 0.054

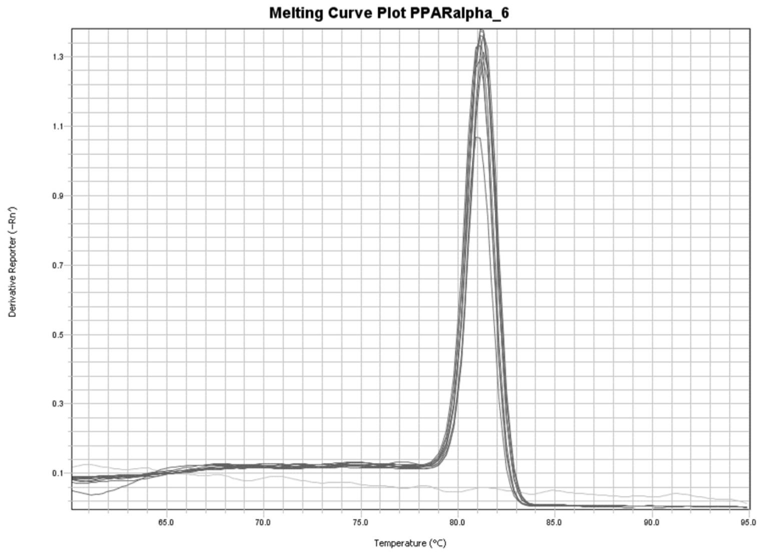

Gene	*Tnfα*
MGI/NCBI ID	104798/21926
Forward primer	GCTTGTCACTCGAATTTTGAGA
Reverse primer	ATGTCTCAGCCTCTTCTCATTC

Job name	Job 1: FTNF-alpha_4
Species	Mouse (Mus musculus)
Assembly	GRCm38
Search type	BLASTN (NCBI Blast)

Download results file   New job

Results table

Show All entries   Show/hide columns (2 hidden)

Genomic Location	Overlapping Gene(s)	Orientation	Query start	Query end	Length	Score	E-val	%ID
17:35201727-35201748 [Sequence]	Tnf	Reverse	1	22	22 [Sequence]	44.0	0.002	100.00 [Alignment]
15:102735245-102735262 [Sequence]		Reverse	5	22	18 [Sequence]	36.1	0.51	100.00 [Alignment]
9:114945435-114945452 [Sequence]		Reverse	1	18	18 [Sequence]	36.1	0.51	100.00 [Alignment]

Job name	Job 2: RTNF-alpha_4
Species	Mouse (Mus musculus)
Assembly	GRCm38
Search type	BLASTN (NCBI Blast)

Download results file   New job

Results table

Show All entries   Show/hide columns (2 hidden)

Genomic Location	Overlapping Gene(s)	Orientation	Query start	Query end	Length	Score	E-val	%ID
17:35200876-35200897 [Sequence]	Tnf	Forward	1	22	22 [Sequence]	44.0	0.002	100.00 [Alignment]
X:82477547-82477562 [Sequence]		Reverse	5	20	16 [Sequence]	32.1	8.0	100.00 [Alignment]
X:82505803-82505818 [Sequence]		Reverse	5	20	16 [Sequence]	32.1	8.0	100.00 [Alignment]

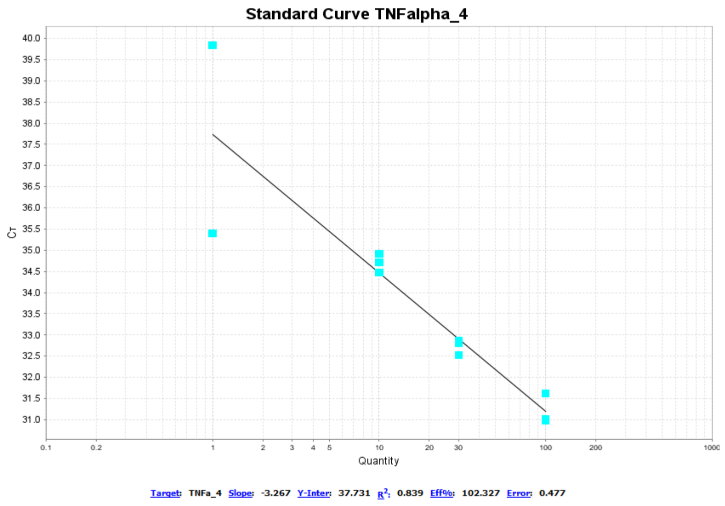

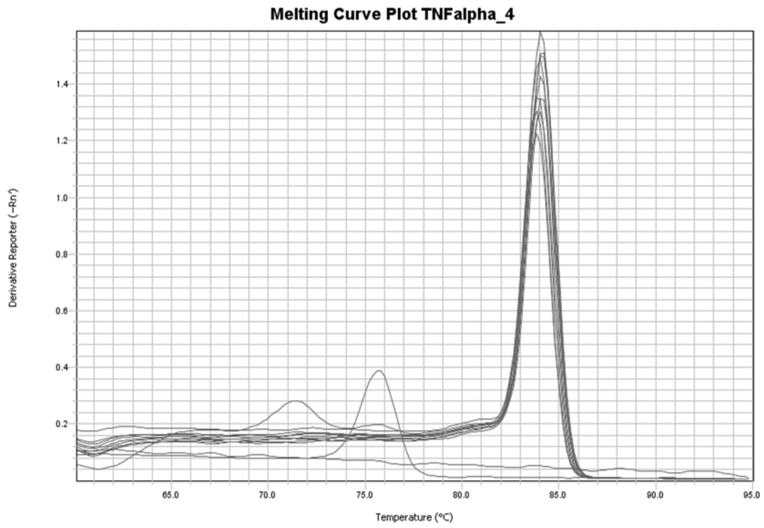

Supplement: Supplementary file 1 [file ijms-24-09691-s001.zip › ijms-2389661-supplementary.pdf]
